# Driving Behavior Based Relative Risk Evaluation Using a Nonparametric Optimization Method

**DOI:** 10.3390/ijerph182312452

**Published:** 2021-11-26

**Authors:** Qiong Bao, Hanrun Tang, Yongjun Shen

**Affiliations:** School of Transportation, Southeast University, Nanjing 210096, China; baoqiong@seu.edu.cn (Q.B.); 220183028@seu.edu.cn (H.T.)

**Keywords:** driving behavior, relative risk, data envelopment analysis, area method

## Abstract

Evaluating risks when driving is a valuable method by which to make people better understand their driving behavior, and also provides the basis for improving driving performance. In many existing risk evaluation studies, however, most of the time only the occurrence frequency of risky driving events is considered in the time dimension and fixed weights allocation is adopted when constructing a risk evaluation model. In this study, we develop a driving behavior-based relative risk evaluation model using a nonparametric optimization method, in which both the frequency and the severity level of different risky driving behaviors are taken into account, and the concept of relative risk instead of absolute risk is proposed. In the case study, based on the data from a naturalistic driving experiment, various risky driving behaviors are identified, and the proposed model is applied to assess the overall risk related to the distance travelled by an individual driver during a specific driving segment, relative to other drivers on other segments, and it is further compared with an absolute risk evaluation. The results show that the proposed model is superior in avoiding the absolute risk quantification of all kinds of risky driving behaviors, and meanwhile, a prior knowledge on the contribution of different risky driving behaviors to the overall risk is not required. Such a model has a wide range of application scenarios, and is valuable for feedback research relating to safe driving, for a personalized insurance assessment based on drivers’ behavior, and for the safety evaluation of professional drivers such as ride-hailing drivers.

## 1. Introduction

Every year, around 1.35 million people die as a consequence of road crashes worldwide [1]. The huge costs in health services, and the added burden on public finances due to road traffic injuries and fatalities, representing approximately 1–3% of GDP in most countries, have become increasingly socially unacceptable [2]. On the other hand, road crashes, previously regarded as random, unavoidable ‘accidents’, have been increasingly identified as a preventable public health problem due to the development of a better understanding of the nature of crashes over the past decades [3,4,5,6]. It is widely understood that drivers’ risky or abnormal behaviors are highly related to road crashes. Studies have shown that over 90% of all road crashes are caused fully or in part by human error [7]. It is therefore of great importance to estimate each driver’s risk before his/her risky driving behaviors lead to a crash. Once risk is evaluated, drivers can better understand their current driving ability, and various interventions can be taken to improve their driving performance. For instance, a dynamic adjustment of vehicle insurance premiums can be applied, and a “reward system” can be introduced to encourage safer driving [8,9].

Over the last decades, many researchers have investigated drivers’ behaviors by using naturalistic driving data [10,11,12,13,14]. For example, Jun, Ogle and Guensler tested in-vehicle equipment that incorporates GPS (Global Positioning System) and OBD (On Board Diagnostics) to gather drivers’ naturalistic driving data, related to speed, acceleration and deceleration, and they were used to predict drivers’ crash risk. The study found that a relationship does not necessarily exist between driving behavior and driver personality characteristics. However, in general, speed, acceleration and braking have been associated with crash involvement [10]. Bagdadi and Varhelyi adopted accelerometers to collect driver’s naturalistic driving data and proposed a new method to predict acceleration- and braking- related crash risks [11]. Şimşek et al. highlighted the benefits of using GPS data to construct a velocity-time model as the basis for studying driving behavior [12]. Castignani et al. proposed the use of a mobile phone as a low-cost platform for monitoring daily driving behavior [13]. Grimberg, Botzer and Musicant further presented a comparative review of the advantages and disadvantages of using smartphone and in-vehicle data acquisition systems in naturalistic driving studies [14]. Another commonly used method for the collection of driving behavior data is to use driving simulators, which provide a safe and cost-effective means of collecting field data and allow drivers to make decisions and take actions that can have potentially dangerous consequences [15,16,17,18,19,20,21].

By collecting driving behavior data, risky and abnormal driving events can be identified, and research on risk evaluation and safety intervention can be conducted [22]. In this respect, Toledo, Musicant and Lotan proposed a driving risk index based on drivers’ behavioral data, collected by an in-vehicle data recorder. Different risky driving behaviors were taken into account, such as speeding, emergency braking, and sharp lane changing. Each of the behaviors was weighted according to the duration of the behavior [23]. Boquete et al. considered factors such as driving time and the occurrence frequency of risky driving events, and proposed a model of drivers’ premium to facilitate the risk evaluation of driving behaviors [24]. Şimşek et al. provided a conceptual framework for an evaluation of safety intervention and operational performance through monitoring quantitative driver performance measures, including speed violations and vehicle idle duration [12]. Musicant, Bar-Gera and Schechtman utilized an onboard data recorder to gather drivers’ risky behaviors during their three years’ natural driving, and the characteristics of their risky driving behaviors over time as well as the influence of their trip duration were explored [25]. Castignani et al. proposed a fuzzy system to identify risky driving events such as speeding, sharp acceleration, emergency braking, and sharp turns. Drivers were scored from 0 (worst) to 100 (best) by collecting the frequency of risky driving events over a predefined distance [13]. Ellison, Bliemer and Greaves evaluated drivers’ risky behaviors based on the maximum, average, minimum, and standard deviations of vehicle speed, acceleration, and deceleration for some unsafe driving events. A fixed value, obtained from the literature was used to quantify the risk caused by speed, acceleration and deceleration, respectively, and the individual risky behavior scores were further weighted by the contribution of each behavior to crash risk, which was also fixed, so as to obtain a total composite score [26]. Hong, Chen and Wu proposed a new approach for driver risk assessments, by using a comparison with a “standard” driver, created based on the frequency of risky driving behaviors of a large number of different drivers, including the frequency of sharp acceleration, emergency braking and sharp turns [27].

Although a number of studies concerning driving behavior analyses have been conducted over recent years, some concerns still need to be tackled. First, when estimating the risk of drivers based on their various risky driving behaviors, considering the frequency of these risky driving behaviors within a time dimension—as many of the aforementioned studies have—is important, but not enough. The severity level of the risky driving behaviors should also be taken into account. However, they have frequently been neglected in previous studies. Second, when developing a risk evaluation model based on drivers’ multiple risky driving behaviors, an absolute risk factor is often used to relate each behavior to its corresponding risk, which is, however, unknown in reality and presents great uncertainty with regard to estimations. In this study, we develop a driving behavior-based relative risk evaluation model, in which both the frequency and severity level of risky driving behaviors are considered, and the concept of relative risk instead of absolute risk is proposed. To do so, a data envelopment analysis (DEA) [28] is employed, which is a nonparametric optimization method for measuring the relative efficiency for a set of entities, referred to as decision making units (DMUs), such as drivers. Based on the identification of various risky driving behaviors from the naturalistic driving data of multiple drivers in their different trips, the proposed model is applied to assess the overall risk relative to distance travelled of an individual driver during a specific driving segment in comparison to other drivers on other segments. The results are further compared with those from the absolute risk evaluation.

The rest of this paper is organized as follows: Section 2 introduces the driving behavior-based relative risk evaluation model based on the mechanism of data envelopment analysis. The results from the model are presented via a case study in Section 3 and further compared with those from the absolute risk evaluation. Section 4 discusses the advantages and limitations of the proposed model. Section 5 summarizes some important findings from this study and also provides directions for future research.

## 2. Methodology

To assess the crash risk of a driver based on his/her driving behavior, in this study we develop a driving behavior-based relative risk evaluation model, using a nonparametric optimization method. In this model, both the frequency and the severity level of different risky driving behaviors are considered, and the concept of relative risk instead of absolute risk is proposed.

### 2.1. Data Envelopment Analysis

The DEA method was first proposed by Charnes, Cooper and Rhodes in 1978 [28]. Following its proposal, the DEA has been recognized as a powerful analytical research tool for modeling operational processes in terms of performance evaluations and benchmarking [29,30,31,32]. As a nonparametric method, DEA applies mathematical optimization techniques to estimate the relationship between multiple inputs and multiple outputs related to a set of DMUs. During these years, a number of different formulations were proposed in the DEA context, the best-known of which is probably the Charnes–Cooper–Rhodes (CCR) model. Specifically, suppose that there are *n* DMUs. Each DMU has *m* inputs and *s* outputs as follows:

$$x_{k}={\left(x_{1k},x_{2k},\ldots ,x_{ik},\ldots ,x_{mk}\right)}^{T}>0,\;k=1,\;2,\;\ldots ,\;n$$

$$y_{k}={\left(y_{1k},y_{2k},\ldots ,y_{rk},\ldots ,y_{sk}\right)}^{T}>0,\;k=1,\;2,\;\ldots ,\;n$$

where $x_{k}$ represents the set of inputs of the *k*th DMU, and $x_{ik}$ is the value of the ith input for the kth DMU. $y_{k}$ represents the set of outputs of the kth DMU, and $y_{rk}$ is the value of the rth output for the kth DMU.

The relative efficiency of a DMU is defined as the ratio of its total weighted output to its total weighted input, between zero and the unity. Mathematically, the efficiency score of the $k_{0}\mathrm{th}$ DMU is obtained by solving the following constrained optimization problem [28]:

$$\max h_{k0}=\frac{\sum_{r=1}^{s}u_{r}y_{rk0}}{\sum_{i=1}^{m}v_{i}x_{ik0}}$$

(1) $$s.t.\{\begin{array}{c} \frac{\sum_{r=1}^{s}u_{r}y_{rk}}{\sum_{i=1}^{m}v_{i}x_{ik}}\leq 1,\;k=1,\;2,\;\ldots ,\;n\\ v={\left(v_{1},\;v_{2},\;\ldots ,\;v_{m}\right)}^{T}\geq 0\\ u={\left(u_{1},\;u_{2},\;\ldots ,\;u_{s}\right)}^{T}\geq 0 \end{array}$$

where $u_{r}$ is the weight given to output *r*, and $v_{i}$ is the weight given to input *i*. This fractional program is computed separately for each DMU to determine its optimal input and output weights. In other words, the weights in the objective function are automatically selected from the model with the purpose of maximizing the value of the $k_{0}\mathrm{th}$ DMU’s efficiency ratio and also respecting the less-than-or-equal-to-one constraint for all the DMUs.

The model described above can be further simplified when inputs or outputs are constant. It then becomes the following constrained optimization problem, which is known as the CCR model with constant inputs [31].

$$\max h_{k0}=\overset{s}{\underset{r=1}{\sum }}u_{r}y_{rk0}$$

(2) $$s.t.\{\begin{array}{c} \overset{s}{\underset{r=1}{\sum }}u_{r}y_{rk}\leq 1,\;k=1,\;2,\;\ldots ,\;n\\ u={\left(u_{1},\;u_{2},\;\ldots ,\;u_{s}\right)}^{T}\geq 0 \end{array}$$

The *n* DMUs are, in this problem, evaluated by combining *s* different outputs (or indicators), with higher values indicating better performance, while the inputs of each DMU in Model (1) are all assigned a value of unity. Based on Model (2), the best-performing DMUs are found to be those with a relative index score of one, while the others underperform.

The attractive features of DEA, relative to the other methods are as follows: (1) It provides a new way of combining multiple inputs and/or multiple outputs simultaneously, without resorting to a priori knowledge concerning the input and/or output weights. (2) The inputs and outputs used in the model can be expressed in different units of measurement. In other words, the preliminary normalization of raw data is not required. (3) It assesses the relative performance of a particular unit by comparing it against all other units, and its final score is measured relative to the best observed performance, which is particularly suitable for this study.

### 2.2. A Driving Behavior-Based Relative Risk Evaluation Model

To evaluate the relative risk of a driver, risky driving behaviors should firstly be distinguished from safe behaviors. Here, a driving behavior with data exceeding a certain threshold value (e.g., the speed limit of a road section) is defined as a risky driving behavior. Such a driving state will bring a certain degree of risk to the driver and other surrounding road users as well. Furthermore, different severity levels of risk should be taken into account when a specific risky driving behavior is identified. For instance, the risk of speeding over the speed limit by 10 and 50% should be different. In addition, for those behaviors not identified as risky driving behaviors, we should not regard them as risk-free, so a constant risk value of 1 is set in this study.

Assuming that different drivers’ driving behavior data are collected based on their naturalistic driving, the risk of various risky driving behaviors during a certain time period is calculated as follows. First, a threshold value is set for each type of driving behavior so as to identify risky driving behaviors. Taking speed as an example, the speeding threshold is considered to be the speed limit value of the road section on which the driver is driving. Moreover, the speeding magnitude changes dynamically. To consider different severity levels of risky driving behaviors, it is theoretically possible to treat each magnitude exceeding the threshold value as a separate evaluation category. A severity weight $W_{ij}$ can then be set for each category, where i represents various risky driving behaviors (*i* = 1, 2, …, *p*), and j represents the evaluation categories (*j* = 1, 2, …, *q*).

The severity weight $W_{ij}$ is used at time *t* to establish the relationship between the driving behavior data exceeding the threshold and the corresponding risk $R_{ij}^{t}$. At a higher severity rate, a higher severity weight will be assigned, resulting in a higher risk. After this, the risk of each risky driving behavior for all unit times is calculated separately to obtain the total risk of a specific risky driving behavior. Taking speeding as an example, suppose that the speed data exceeding the speed limit has q evaluation categories during one trip. Within unit time Δt, the speed $V^{t}$ ($V^{t}$ indicates the speed at time *t*) remains unchanged, and when the predefined speed limit value is exceeded, a severity weight $W_{1j}$ (*i* = 1 represents speeding) is then used to establish the relationship between the area $V^{t}\Delta t$ corresponding to the speed exceeding the speed limit and the risk $R_{1j}^{t}$, that is, $R_{1j}^{t}=W_{1j}\times V^{t}\Delta t$. The risk corresponding to a speed not exceeding the speed limit at time *t* is $R_{1j}^{t}=1$. By accumulating the risk associated with speeding during the whole trip, the total risk for speeding can be obtained, and the risk relative to distance travelled can be computed by dividing it by the road section distance *L*. In the same way, the total driving risk and the risk relative to distance travelled of all other risky driving behaviors can be calculated.

To evaluate the driver’s overall risk during a trip, the contribution of each risky driving behavior to the final risk score should be taken into account. Therefore, another weight, denoted by $v_{1}$, $v_{2}$, …, $v_{p}$, should be assigned to the total risk of each risky driving behavior during the trip.

Thus, we develop the driving behavior-based relative risk evaluation model as described in the following section. The objective function of the model is to minimize the overall risk score of the DMU under study, which is the weighted sum of the total risk of various risky driving behaviors, as shown in (3). The constraints of the model are: (1) The overall risk score of all DMUs given the weights for the DMU under study should be no less than the benchmark value of 1. In other words, the DMU with an overall risk score equal to 1 is the safest DMU among all DMUs. (2) The severity weights $W_{ij}$, are variables and should be arranged according to the extent to which the driving behavior data exceeds the corresponding threshold. The more severely the behavior data scores over the threshold value, the higher the risk of this behavior. Accordingly, the value of the severity weight is greater. Since the risk of safe driving behavior is set to 1, the lowest value of the severity weight should be greater than 1. (3) The values of all weight variables should be greater than or equal to 0.

$$\min r_{k0}=\overset{p}{\underset{i=1}{\sum }}\left[v_{i}\times \left(\overset{q}{\underset{j=1}{\sum }}W_{ij}\times x_{ijk0}\right)\right]$$

(3) $$s.t.\{\begin{array}{c} \overset{p}{\underset{i=1}{\sum }}\left[v_{i}\times \left(\overset{q}{\underset{j=1}{\sum }}W_{ij}\times x_{ijk}\right)\right]\geq 1,\;k=1,\;2,\;3,\;\ldots ,n\\ W_{iq}>\ldots >W_{i2}>W_{i1}>1,\;i=1,\;2,\;3,\ldots ,p\\ v={\left(v_{1},\;v_{2},\;\ldots ,\;v_{p}\right)}^{T}\geq 0 \end{array}$$

In the above-described model, a driver that drives on a certain road segment can be treated as a decision-making unit $DMU_{k}$, where $x_{ijk}$ represents the summation of the area corresponding to the jth class of the ith risky driving behavior relative to distance travelled of the kth DMU. $W_{ij}$ represents the severity weight of the jth class of the ith risky driving behavior. $v_{i}$ denotes the behavior category weight, which is used to represent the relative contribution of the ith risky driving behavior to the overall risk. $\sum_{i=1}^{p}\left[v_{i}\times \left(\sum_{j=1}^{q}w_{ij}\times x_{ijk}\right)\right]$ is the overall risk score of the *k*th DMU. A higher score indicates a greater relative risk. Thus, a DMU that obtains a score of 1 is the safest DMU under study.

Since both the severity weight and the behavior category weight are variables here, Model (3) is nonlinear. Consequently, variable transformation is applied to convert this model into a linear one. More specifically, a new variable, $U_{ij}$, is introduced as the product of these two weight variables, that is, $U_{ij}=v_{i}\times w_{ij}$. Thus, a linear model is constructed as follows:

$$\min r_{k0}=\overset{p}{\underset{i=1}{\sum }}\overset{q}{\underset{j=1}{\sum }}U_{ij}\times x_{ijk0}$$

(4) $$s.t.\{\begin{array}{c} \overset{p}{\underset{i=1}{\sum }}\overset{q}{\underset{j=1}{\sum }}U_{ij}\times x_{ijk}\geq 1,\;k=1,\;2,\;3,\;\ldots ,n\\ U_{iq}>\ldots >U_{i2}>U_{i1}>v_{i},\;i=1,\;2,\;3,\ldots ,p\\ v={\left(v_{1},v_{2},\;\ldots ,\;v_{p}\right)}^{T}\geq 0 \end{array}$$

## 3. Application and Results

### 3.1. A Case Study

In the naturalistic driving experiments, the RACELOGIC’s third-generation GPS data collector VBOX3i was used as the driving behavior data collection device, which was installed in the vehicle of the drivers who participated in the experiments. In total, 14 voluntary drivers were enrolled, and they were asked to drive around Nanjing city for the day as they usually would. Thereafter, basic data including time (s), longitude (degree), latitude (degree), speed (km/h), acceleration ($m/s^{2}$), and deceleration ($m/s^{2}$) were gathered after performing necessary data cleaning and coordinate transformation procedures, and the API (Application Programming Interface) of the Gaode Map was used to connect the collected data to location. To avoid the influence of certain external factors (e.g., traffic signal control) on drivers’ instinctive behavior, 60 driving segments on straight road sections with continuous positioning, a constant speed limit, and for a distance of no less than 0.8 km were extracted and are treated as 60 DMUs in this study.

The risky driving behaviors considered in this study include speeding, sharp acceleration and emergency braking, which are defined as characteristics of a vehicle that demonstrates speed or acceleration exceeding a certain threshold value. With respect to speeding, the speed limit of each road section is used as the threshold in this study. For acceleration and deceleration, $3\;m/s^{2}\;$ and $2\;m/s^{2}$ are set as the threshold of sharp acceleration and emergency braking, respectively, as many studies have found that drivers with an acceleration exceeding $3\;m/s^{2}$ or with a deceleration greater than $2\;m/s^{2}$ demonstrated a significantly higher crash incidence in crash statistics [33,34,35].

Furthermore, to take the different severity levels of each risky driving behavior into account, we adopted the K-Means clustering method [36] to generate the number of evaluation categories. That is, for each of these three risky driving behaviors, when the value within the unit time of time *t* exceeds the corresponding threshold, the magnitude exceeding the threshold (denoted as ΔV, ΔA, and ΔB for speeding, sharp acceleration, and emergency braking, respectively) is recorded. Then, the K-Means clustering analysis is performed to classify the listed magnitudes. To guarantee that the grouped samples are generally uniformly distributed, in this case study, each of the three driving behaviors that exceed the aforementioned threshold values are grouped into three clusters. The results are shown in Table 1. Of course, other numbers of the clusters can also be considered.

**Figure 1:**
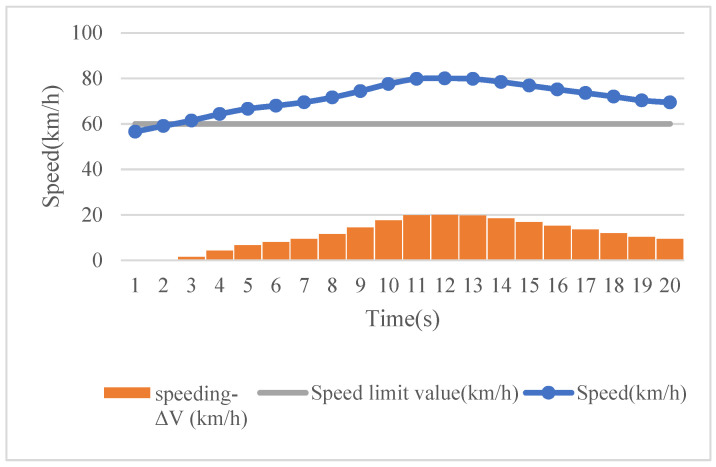
The magnitude of speeding—the area method.

Next, we propose the usage of the area method to calculate the risk of different driving behaviors during one driving segment. Taking speed as an example, 20 s of naturalistic driving data has been used to illustrate the method. As shown in Figure 1, each rectangle in this figure represents the area formed by the speed exceeding the speed limit value per unit time (i.e., 1 s here). In such a time unit, we assume that the speed remains unchanged, and when the speed limit value is exceeded, the result is classified into one of the speeding clusters presented in Table 1. Then, by multiplying it with a corresponding severity weight, we are able to represent the speeding risk at the given time unit, and the total risk of speeding during this 20 s time period is identified through the accumulated risk at each time unit. Thus, risk quantification is adapted from the use of one dimension to two dimensions, considering not only the occurrence frequency of the risky driving events, but also their severity levels. With regard to a driving behavior value lower than the threshold, a constant risk value of 1 is set.

**Figure 2:**
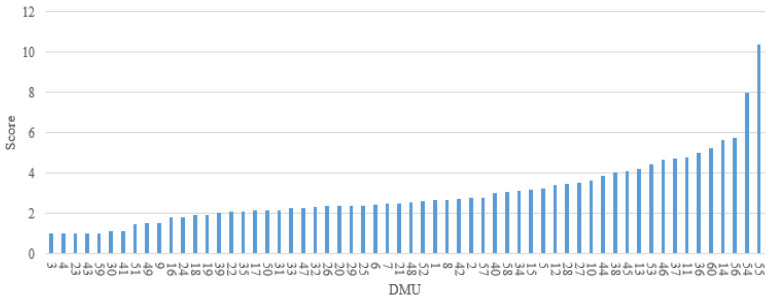
Overall risk scores and corresponding ranking of 60 DMUs.

The proposed Model (4) can be applied to evaluate the relative risk of the 60 DMUs selected in this study. Their overall risk scores and the relative safety ranking are presented in Figure 2. The DMU that received a score of 1 represents the driver with the safest driving behavior on the given road segment among all 60 DMUs, namely, DMUs 3, 4, 23, 43 and 59. While DMUs 54 and 55 obtain an overall risk score of higher than 8, which implies that the risk of the drivers driving on these two road segments is at least 8 times higher than the others under evaluation.

**Figure 3:**
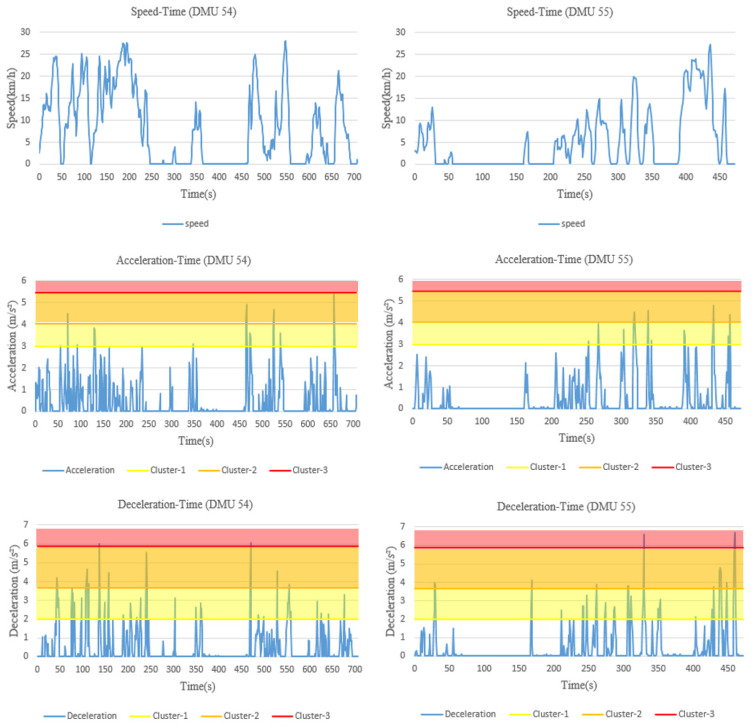
The driving behavior data of DMUs 54 and 55.

To further understand the reasons behind such a high relative risk score received by these two DMUs, the original driving behavior data of the drivers on these two road segments are retrieved, presented in Figure 3.

**Figure 4:**
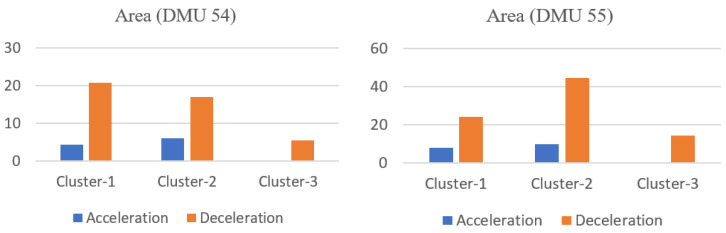
The area accumulation with respect to acceleration and deceleration for DMUs 54 and 55.

The speed limit of both road sections is 50 km/h, and we can see that neither of these two DMUs exceed the speed limit during driving. Regarding the other two risky driving behaviors, however, both of the DMUs demonstrate certain moments in which their acceleration or deceleration values exceed the second severity level of sharp acceleration and the third severity level of emergency braking. Their risky behavior areas that score above the thresholds are shown in Figure 4. It can be seen that the area of emergency braking is relatively large for both DMUs (DMU 55 is even larger), which contributes the most to their overall risk, and results in higher overall relative risk relative to distance travelled compared to the others.

### 3.2. A Comparison with the Absolute Risk Evaluation

One of the most important contributions of the proposed model described in Section 2 is to manage the challenge of the absolute risk of each driving behavior being unknown. In other words, the severity weights $W_{ij}$ that were used to convert each behavior into its risk should not be a fixed value, but a variable. We can ensure that the larger the degree by which behavior data exceeds the threshold value, the higher the risk of this behavior, and accordingly, the greater the severity weight. As a result, in this study, we developed a driving behavior-based relative risk evaluation model, in which the concept of relative risk instead of absolute risk is proposed. To verify the results obtained from this model, we establish another model to evaluate the absolute risk of these 60 DMUs by referring to the fixed risk values provided in the literature. Specifically, some studies have investigated the relationship between speed and crash risk. One of the most authoritative studies was conducted by Kloeden and McLean in 1997 on roads with a speed limit of 60 km/h in Australia [37]. The result from their research concerning the relationship between speeding and risk is summarized in Table 2. The results derived by Bagdadi and Varhelyi [11] are utilized for acceleration and deceleration, as shown in Table 3.

**Figure 5:**
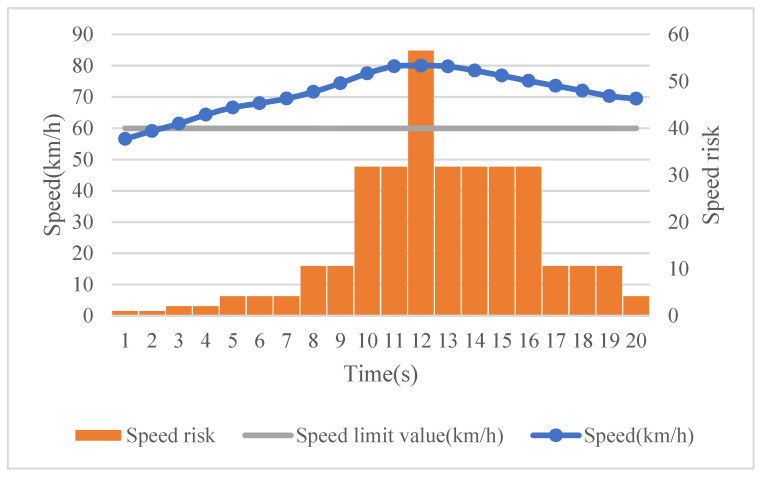
The absolute risk of speeding—the area method.

Using the fixed risk values given in Table 2 and Table 3, the relationship between the driving behavior data exceeding the threshold values and the corresponding risk can be established in unit time at time *t*. The area method can then be applied to calculate the absolute risk of various driving behaviors during a trip. Speed is considered as an example and 20 s of naturalistic driving data are used to illustrate the method (shown in Figure 5). Each rectangle in this figure represents the risk posed by the speed per unit time according to Table 2. Thus, the total risk of speeding during this 20 s time period represents the accumulation of the risk at each time unit.

By obtaining the total risk for all three risky driving behaviors at each road segment under consideration, the following absolute risk evaluation model can be formulated:

$$\min r_{1}=\overset{3}{\underset{i=1}{\sum }}v_{i}\times x_{i1}^{\prime }$$

(5) $$s.t.\{\begin{array}{c} \overset{3}{\underset{i=1}{\sum }}v_{i}\times x_{ik}^{\prime }\geq 1,\;k=1,\;2,\;3,\;\ldots ,\;60\\ v={\left(v_{1},\;\;v_{2},\;v_{3}\right)}^{T}\geq 0 \end{array}$$

where $x_{ik}^{\prime }$ represents the total risk corresponding to the  ith driving behavior relative to distance travelled of the kth DMU. $v_{i}$ denotes the behavior category weight, which indicates the relative contribution of the ith risky driving behavior to the overall risk. Since no severity weight variable is considered, we use a linear programming model, and solutions can be obtained using Lingo software directly.

**Figure 6:**
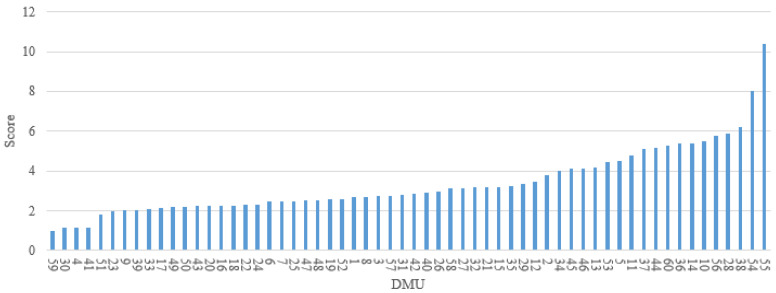
Overall risk scores and corresponding ranking of 60 DMUs using fixed risk values.

Applying the same naturalistic driving data to Model (5), we obtain the overall risk scores of the same 60 DMUs and their ranking, shown in Figure 6.

**Figure 7:**
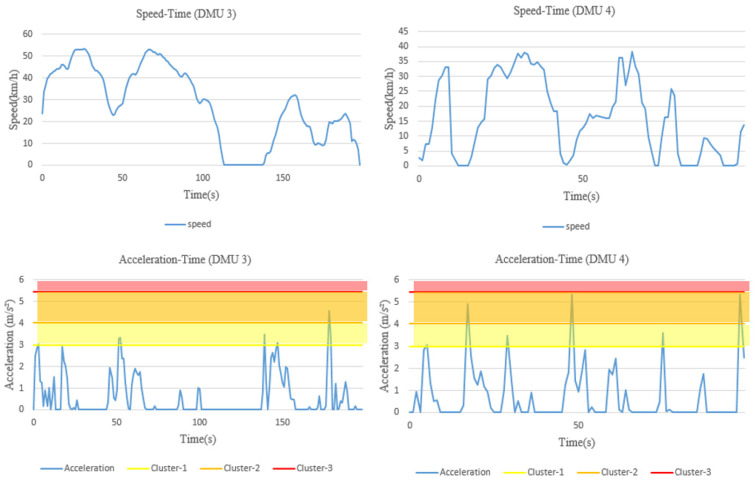

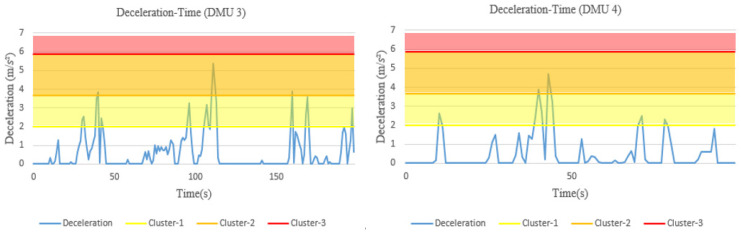
The driving behavior data of DMUs 3 and 4.

**Figure 8:**
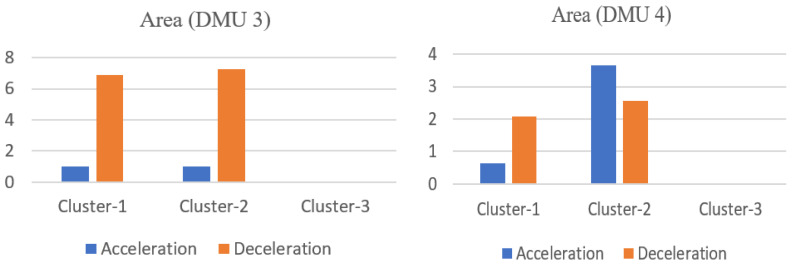
The area accumulation with respect to acceleration and deceleration for DMUs 3 and 4.

By comparing the results from the two models, a high correlation coefficient with respect to the overall risk scores is found, which is 0.933. DMUs 4 and 59 obtain a relatively lower overall risk score from both models, implying that they are better-performers among all others, regardless of which model is applied. Conversely, DMUs such as 54 and 55 always receive a higher risk value and rank at the bottom of all the DMUs involved in the evaluation. However, there are also some variations. The DMU with the largest difference in ranking is DMU 3, obtaining the lowest overall risk score of 1 from Model (4), but ranks in the middle by Model (5). The original data of this DMU together with DMU 4 (a relatively good performer from both models) are shown in Figure 7. The risky behavior areas above the thresholds of these two DMUs are shown in Figure 8.

The speed limit of both road sections is 60 km/h. It can be seen that both drivers do not exceed the speed limit during the trip. Although sharp acceleration and emergency braking occurs from time to time for both DMUs, the occurrence frequency relative to distance travelled is much lower than for the high-risk DMUs shown in Figure 3, and neither demonstrates acceleration and deceleration values exceeding the third severity level. It is therefore reasonable to treat both DMUs 3 and 4 as better-performers. Nevertheless, since the fixed risk values are assigned directly to the behavior data in the absolute risk evaluation model, and they increase exponentially in relation to the extent to which the behavior data exceeds the different levels of the threshold, especially for deceleration (see Table 3), DMU 3, for which deceleration performance is slight worse than that of DMU 4, obtains a much higher overall risk score (2.727 vs. 1.140) and a worse ranking from this model, although its acceleration performance is found to be better than DMU 4.

## 4. Discussion

Having developed and applied the driving behavior-based relative risk evaluation model and having compared the results with those from the absolute risk evaluation model, we are able to review the whole modeling process and discuss its main advantages and limitations. First, when estimating the risk of drivers based on their various risky driving behaviors, not only the occurrence frequency of these risky driving behaviors, but also their severity levels should be taken into account. In this study, the area method is proposed to collect the risky driving behavior data over the threshold values along the time dimension. Thus, the severity levels of each risky driving behavior are considered in the evaluation. Second, given the fact that the absolute risk associated with each driving behavior is usually unknown or estimated with a large degree of uncertainty, a severity weight $W_{ij}$ is introduced in this study to convert each behavior into its risk, which is not a fixed value, but a variable with its value increasing in relation to the degree to which the behavior data exceeds the threshold value. Third, to evaluate a driver’s overall risk relative to distance travelled, the contribution of each risky driving behavior to the final risk score should be different and driver-dependent. Accordingly, another variable, i.e., a behavior category weight $v_{i}$ is assigned, and a nonparametric optimization model—employing a data envelopment analysis (DEA) for relative efficiency evaluation—is developed. By minimizing the overall risk score relative to distance travelled of the driver under study and taking all other drivers into account, both the severity weights and the behavior category weights are derived simultaneously (in cross-product form, i.e., $U_{ij}$), and the relative risk of each driver instead of his/her absolute risk is obtained. Consequently, it provides a valuable solution to the difficulties of behavior-based risk evaluation in quantifying the relationship between each risky driving behavior and crash risk on the one hand, and in estimating the contribution of various risky driving behaviors to the overall crash risk on the other. In addition, the nonparametric nature of the model means that it is a ‘data-oriented’ technique and assumptions about the functional form of the optimal input-output relations are not necessary, which is beneficial as they are often complex or even unknown in the real world situation [38].

Regarding the comparison with the absolute risk evaluation model adopted in this study, although both of the models (Models (4) and (5)) use the idea of DEA, their mechanisms are fundamentally different. In Model (4), the input data includes the accumulated area of each risky driving behavior in different clusters. The severity weights that are used to convert each behavior into its risk, are treated as (part of) the decision variables of the model. Thus, as long as the behavior severity clusters are determined, the input values of the model can be derived from the original driving behavior data directly. Whereas in Model (5), the fixed risk values should be predefined for each cluster, and they are used as the input of the model. Hence, even if the same clusters are applied, the results obtained from these two models would be different. In other words, Model (4) is not a special case of Model (5), and they are not interchangeable. In general, Model (4) is preferred in practical applications, as it avoids the absolute risk quantification for all kinds of risky driving behaviors. Meanwhile, in contrast to the fixed number of clusters considered in Model (5), the clusters used in Model (4) are derived from the original driving behavior data. Therefore, the number can be determined based on practical needs, and a sensitivity analysis can be conducted to reveal the impact of adopting a different number of clusters on the relative risk evaluation.

However, there are also some limitations in this study. (1) The models are restricted to the in-vehicle device used for driving behavior data collection; only three behavioral indicators—speed, acceleration, deceleration—are considered for risk evaluation in this study, which may not be sufficient to represent the overall risk of a driver. During the actual driving process, drivers may be influenced by overtaking behavior, following behavior and lane changing behavior of the surrounding vehicles. These behavioral data could be acquired by using video acquisition equipment either installed in vehicle or at roadsides. Although they are important in risk evaluation, they are not included in this study due to data unavailability. (2) In the naturalistic driving experiments, drivers may not encounter the same traffic environment, even if they drive through the same road section. It implies that the drivers who are identified as high-risk from the model may have encountered events that were not experienced by other drivers. For those underperforming drivers, lessons from the better-performing drivers with respect to safer driving behaviors may not be learned directly. (3) By applying the proposed model, 60 DMUs have been evaluated in the case study, including 60 different driving segments, conducted by 14 drivers. In other words, a driver may be considered within different units of evaluation when multiple segments from his/her driving sessions are selected. This does not present a problem from the perspective of model application, as the same driver may perform differently in different trips and can be ranked by his/her driving performance in these trips. In this way, however, the impact of a driver’s personal characteristics (e.g., gender, age, personality, historical crashes) on his/her risky driving behaviors cannot be assessed.

## 5. Conclusions and Future Research

Given the fact that road crashes are rare and random events, research on drivers’ naturalistic driving behavior data is widely recognized as a promising direction for risk evaluation, and great efforts have been made in this regard over the past decades. However, a uniform and methodological framework has not yet been successfully established. The difficulties encountered in this research topic mainly concern two aspects: (1) how to quantify the relationship between each risky driving behavior and crash risk, and (2) how to estimate the contribution of various risky driving behaviors to the overall risk. To properly address these issues, we developed, in this study, a driving behavior-based relative risk evaluation model by using a nonparametric optimization method. More specifically, by adopting an area method, both the occurrence frequency and the severity level of different risky driving behaviors are taken into account. After which a severity weight and a behavior category weight are introduced to convert each behavior into a value for its risk and to identify the contribution of various risky driving behaviors to the final risk score, respectively. Instead of using constant values that need to be predefined, these weights are treated as decision variables in this study and are determined by applying the nonparametric optimization model. In this way, the absolute risk quantification of all kinds of risky driving behaviors is avoided, and a prior knowledge on the contribution of different risky driving behaviors to the overall risk is not required. Meanwhile, no assumption about the functional form of the optimal input–output relations is required. In the case study, based on the identification of various risky driving behaviors from the naturalistic driving data of multiple drivers in their different trips, the proposed model is applied to assess the overall risk relative to distance travelled of individual drivers. The results are further compared with the ones from the absolute risk evaluation.

The model proposed in this paper has a wide range of application potentials. Individual drivers, by comparing their driving behaviors with others, are able to better understand their own driving performance, and key problems related to safe driving can be diagnosed for each driver separately, and driver-specific improvement strategies can also be formulated. All these results are valuable for feedback related to safe driving, for a personalized insurance assessment based on drivers’ behavior, and for the safety evaluation of professional drivers such as ride-hailing drivers. Moreover, the long-term tracking and analysis of a large number of different drivers’ behaviors will help road safety policy-makers to formulate more reasonable traffic regulations and provide more extensive insights in effective road-safety management.

In future studies more aspects could be investigated. (1) To represent a more enriched picture of behavior-related crash risk, other behavioral indicators such as turning behavior, following behavior, lane changing behavior, etc. should be developed and refined, and the model proposed in this study can be applied directly once indicator data are available. (2) Traffic environment factors that are encountered differently for different drivers, should be taken into account in drivers’ relative risk evaluation. However, since they should be treated as non-discretionary (or non-controllable) variables in the evaluation, new methodological challenges will likely appear when integrating such variables into the existing model. (3) By collecting more drivers’ behavior data, each driver could be treated as a separate DMU, through which the impact of a driver’s personal characteristics on his/her risky driving behaviors can be explored. (4) Apart from the CCR model introduced in this study, the added value of using a large number of other DEA models (such as the BCC model, the additive model, the slacks-based measure of efficiency, and the multiplicative model) can also be investigated in future.

## Figures and Tables

**Table 1:** Clustering results of risky driving behaviors.

Clusters	Speeding-ΔV (km/h)	Sharp Acceleration-ΔA (m/s^2^)	Emergency Braking-ΔB (m/s^2^)
1	0<ΔV≤8.0904	0<ΔA≤1.0298	0<ΔB≤1.6769
2	8.0904<ΔV≤17.7669	1.0298<ΔA≤2.4461	1.6769<ΔB≤3.8668
3	17.7669<ΔV	2.4461<ΔA	3.8668<ΔB

Note: ΔV represents the amplitude exceeding the speed threshold, ΔA represents the amplitude exceeding the acceleration threshold, and ΔB represents the amplitude exceeding the deceleration threshold.

**Table 2:** Risk of speeding under 60 km/h speed limit condition.

Speed (km/h)	Risk
Speed≤ 60	1
60 <Speed≤ 65	2
65 <Speed≤ 70	4.16
70 <Speed≤ 75	10.6
75 <Speed≤ 80	31.81
80 <Speed≤ 85	56.55
Speed> 85	100

**Table 3:** Risk of sharp acceleration and emergency braking.

Acceleration (m/s^2^)	Risk	Deceleration (m/s^2^)	Risk
Acceleration≤ 3 3 <Acceleration≤ 4 4 <Acceleration≤ 5 5 <Acceleration≤ 6 Acceleration> 6	1 3 5 7 9	Deceleration≤ 2 2 <Deceleration≤ 3 3 <Deceleration≤ 4 4 <Deceleration≤ 5 5 <Deceleration≤ 6 Deceleration> 6	1 3 6 12 24 48

## Data Availability

The data presented in this study can be provided by the authors upon request.
